# Role of histone post-translational modifications in atherosclerosis and the therapeutic potential of targeting epigenetic modifiers

**DOI:** 10.3389/fcell.2025.1705966

**Published:** 2025-12-01

**Authors:** Lili Wu, Wei Li, Wei Ye

**Affiliations:** 1 Department of Vascular Surgery, Suzhou Ninth People’s Hospital, Suzhou Ninth Hospital affiliated to Soochow University, Suzhou, Jiangsu, China; 2 Department of Center Laboratory, Kunshan Hospital of Chinese Medicine, Affiliated Hospital of Yangzhou University, Kunshan, Jiangsu, China

**Keywords:** cardiovascular disease, atherosclerosis, histone post-translational modifications, epigenetic, therapeutic targets

## Abstract

Cardiovascular disease (CVD) remains the leading cause of mortality worldwide, with atherosclerosis being the primary pathological substrate underlying most CVD. Epigenetics, defined as a set of regulatory mechanisms that dynamically modulate gene expression patterns or protein functional states through chemical modifications without altering the primary sequence of the genome, has been increasingly recognized as a pivotal driver in the pathogenesis of various diseases. Histone post-translational modifications, such as acetylation, methylation and lactylation, are catalyzed by specific enzymes and are essential for the regulation of gene expression, which in turn influences cellular functions and the progression of diseases. Notably, dysregulation of specific histone modifications is closely associated with the onset and progression of cardiovascular disorders. Accumulating evidence has demonstrated that aberrant histone modifications disrupt vascular cell homeostasis and contribute to atherogenesis by shaping the transcriptional landscape of vascular cells. On the one hand, histone modifications directly influence cellular functions (e.g., endothelial barrier integrity, macrophage lipid phagocytosis, and vascular smooth muscle cell phenotypic switching) and thereby drive atherosclerotic progression. On the other hand, these epigenetic modifications are dynamically modulated by major atherogenic risk factors, including dyslipidemia, pro-inflammatory cytokine release, and hemodynamic stimulation. This review focuses on the multifaceted roles of histone post-translational modifications in mediating vascular dysfunction during atherosclerosis, with an emphasis on the molecular mechanisms linking specific modifications to pathological cellular behaviors. Additionally, we highlight emerging therapeutic strategies targeting histone modification pathways, with the goal of advancing the development of precision diagnostics and interventions for atherosclerosis.

## 1 Introduction

Cardiovascular disease (CVD) represents the leading cause of mortality and disability globally, severely compromising human health and quality of life and imposing an enormous socioeconomic burden on healthcare systems worldwide. Atherosclerosis is the primary etiology and pathological basis underlying most life-threatening CVDs, such as coronary artery disease (CAD) and ischemic stroke (Commodore-Mensah et al., 2021). Therefore, deciphering the intricate pathogenesis and molecular mechanism of atherosclerosis is highly important for prediction and intervention strategies for CVD in advance. Atherosclerosis is a chronic inflammatory vascular disease induced by multiple factors, such as changes in blood flow disorders, dyslipidemia and persistently elevated levels of inflammatory factors. The pathological cascade of atherosclerosis begins with endothelial dysfunction, and many lipids infiltrate the subendothelium. Circulating monocytes are recruited to the damaged endothelium via adhesion molecules and mature into macrophages expressing scavenger receptors, which then bind lipoproteins and differentiate into foam cells. As the disease progresses, foam cells release pro-inflammatory cytokines to recruit additional inflammatory cells, engulf lipids and form atherosclerotic plaques. Concurrently, vascular smooth muscle cells (VSMCs) migrate from the arterial media to the intima, where they undergo a phenotypic switch from a contractile to a synthetic state. In the early stages of plaque development, VSMCs initially form a fibrous cap by producing extracellular matrix proteins, such as collagen and proteoglycans, to stabilize the plaque. However, in advanced lesions, activated macrophages produce enzymes of the matrix metalloproteinases (MMPs) that degrade the ECM, thinning the fibrous cap and rendering the plaque vulnerable to rupture (Libby et al., 2019). The thrombus complex completely or partially blocks the arterial vasculature, resulting in myocardial infarction, stroke or other life-threatening acute clinical events. In addition to lipid deposition and chronic inflammation, accumulating evidence has highlighted the pivotal role of epigenetic changes in regulating the pathophysiological processes of atherosclerosis.

Epigenetic changes dynamically modulate gene expression without altering the genomic sequence and primarily involve DNA methylation, histone post-transcriptional modifications (PTMs), and non-coding RNAs (ncRNAs). Epigenetic modifications dynamically occur in key cell types throughout the initiation and progression of atherosclerotic lesions, including vascular endothelial cells, vascular smooth muscle cells and macrophages (Grimaldi et al., 2015). These cell-specific epigenetic changes directly regulate cellular behaviors, such as EC barrier dysfunction, macrophage foam cell formation and VSMC phenotypic switching, which are pivotal for driving atherosclerotic progression. Notably, epigenetics serves as a critical bridge between genetic predispositions and environmental risk factors, translating external stimuli into stable alterations in gene expression and perpetuating vascular pathology. Among the various epigenetic mechanisms, histone modifications are deeply involved in the development of many diseases, particularly in oncology and cardiovascular biology. Therefore, studying the relationship between cardiovascular diseases and post-translational modifications of proteins may yield valuable novel insights and therapeutic targets.

Histone modifications and their involvement in inflammatory diseases are emerging research fields, but they are attracting increasing attention and growing at a fast pace. Histone post-translational modifications are covalent chemical modifications of amino acid side chains in translated proteins, which are catalyzed by specific enzyme families called “writers”, which add modifications. In addition to histone post-translational modification writers, erasers also exist and remove them, thereby impacting fundamental biological processes such as DNA replication, transcription, and chromosome maintenance. These histone modifications include acetylation, methylation, lactylation, ubiquitination, phosphorylation, and glycosylation (Wang and Wang, 2019). The nucleosome, consisting of 146 base pairs of DNA wrapped around an octamer of core histone proteins, is the basic unit of chromatin and is closely related to the genetic functions of the individual (Shaytan et al., 2015). The core histones include two copies each of H2A, H2B, H3, and H4, which are highly dynamic and susceptible to PTMs, acting as molecular switches to respond to cellular signals, whereas the linker histone H1 stabilizes the higher-order chromatin structure (Rothbart and Strahl, 2014; He et al., 2018). Imbalances in histone modifications can lead to the development of cardiovascular disease (Prasher et al., 2020). Among all histone modifications, acetylation and methylation are the most widely studied in the context of inflammation and atherosclerosis, as they affect gene expression and cellular biological functions. For example, histone acetylation neutralizes the positive charge of lysine residues, reducing electrostatic interactions between histones and negatively charged DNA to open chromatin (Lee et al., 1993). In contrast, histone methylation can either activate or repress transcription, depending on the specific residue and degree of methylation. Modulating histone modifications, particularly through the regulation of enzymes involved in these processes, is a promising therapeutic approach for managing CVD.

Given the important role of histone modifications in atherosclerotic pathogenesis, a comprehensive investigation of the epigenetic mechanisms underlying CVD is essential for advancing our understanding of disease management. In this review, we summarize the latest advances in the field, focusing on key histone modifications during the progression of atherosclerosis, especially the roles of these histone-modifying enzymes in regulating vascular cell functions, such as those of endothelial cells, macrophages, and vascular smooth muscle cells. In addition, we discuss the current prospects and challenges of targeting histone modifications in atherosclerosis, highlighting their roles and translational potential for CVD therapeutic interventions.

## 2 Histone acetylation in atherosclerosis

**FIGURE 1:**
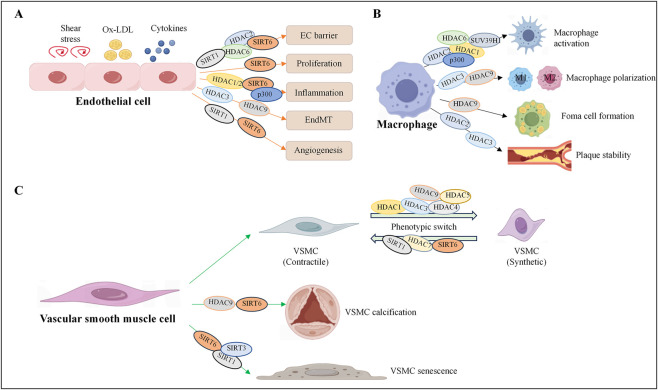
Histone acetylation regulates the fate of vascular cells. Histone acetyltransferases and deacetylases participate in the occurrence and development of cardiovascular diseases by regulating endothelial cell dysfunction **(A)**, macrophage activation, foam cell formation **(B)**, and smooth muscle cell phenotypic transformation **(C)**. HDAC, histone deacetylase; SIRT, sirtuin, histone deacetylase; p300, histone acetyltransferase.

Histone acetylation is the most extensively studied epigenetic modification in the occurrence and development of cardiovascular disease. The enzymes governing histone acetylation are broadly categorized into histone acetyltransferases (HATs), which add acetyl groups, and histone deacetylases (HDACs), which remove them. Acetylation weakens histone‒DNA interactions and loosens the chromatin structure, allowing transcription factors to bind and promote gene expression, whereas deacetylation restores the positive charge of histones and strengthens histone‒DNA binding, leading to chromatin condensation and transcription suppression (Lee et al., 1993). Thus, the dynamic balance between histone acetylation and deacetylation, orchestrated by two opposing enzyme families, serves as a core regulatory switch for controlling gene transcription. HATs are classified into three major families on the basis of their structural and functional characteristics: the GCN5-related N-acetyltransferase family (HAT1, GCN5 and PCAF), the MYST family (MOZ, MORF, HBO1 and TIP60), and the p300/CBP family, which includes p300 and CBP (CREB-binding protein). HDACs are classified into four classes on the basis of their structure and function: Class I HDACs (HDAC1, 2, 3, 8), Class II HDACs, which are subdivided into IIa (HDAC4, 5, 7, 9) and IIb (HDAC6, 10), Class III HDACs (SIRT1-7), and Class IV HDACs, which constitute a small class consisting of only HDAC11 (Park and Kim, 2020). This dynamic regulation of histone acetylation status directly dictates the transcriptional activity of genes controlling inflammation, lipid metabolism, and cell proliferation in vascular cells, thereby establishing histone acetylation/deacetylation as a central epigenetic axis in atherosclerotic pathogenesis (Table 1 for a summary of the HAT/HDAC families involved in atherosclerosis). We illustrate the histone acetylation mechanisms underlying vascular cells (Figure 1).

**TABLE 1:** Histone acetyltransferase and deacetylases in atherosclerosis.

Histone modification enzyme	Class	Name	Targets	Cell type	Function
Histone acetyltransferase (HATs)	GNAT family	GCN5	H3K9、H3K14、H3K18	EC, Mø, VSMC	Enhance TNF-α and IL-6 transcription, and promote M1 polarization
		PCAF	H3K9、H3K14、H4K8	EC, Mø, VSMC	Activate anti-inflammatory gene expression and inhibit endothelial inflammation
	MYST family	MOZ	H3K4、H3K14、H4K5	Mø	Activate CD36 expression and promote foam cell formation
		TIP60	H3K4、H4K5、H4K12	EC	Promote NO production, inhibit apoptosis, and protect endothelial function
	p300/CBP family	p300	H3K27、H3K9、H4K8、H4K16	EC, Mø, VSMC	Enhance endothelial cell chemokine expression and recruit monocytes promote VSMC proliferation and phenotypic switching
		CBP	H3K27、H3K4、H4K12	Mø	Inhibit cholesterol efflux in foam cells
Histone deacetylases (HDACs)	Class I	HDAC1	H3K9、H3K27、H4K12	EC, Mø, VSMC	Promote VSMC proliferation and neointima formation
		HDAC2	H3K9、H3K27、H4K8	VSMC	Enhance M1 polarization and the release of TNF-α and IL-1β
		HDAC3	H3K9、H3K27、H4K16	EC, Mø	Regulate endothelial-mesenchymal transition inhibit macrophage inflammation and intraplaque lipid content
	Class Ⅱa	HDAC4	H3K9、H3K27	VSMC	Activate the p38/MAPK pathway, and promote VSMC proliferation, migration, and vascular calcification
		HDAC5	H3K9、H3K27	EC, Mø	Inhibit angiogenesis activate NF-κB and enhance inflammatory response
		HDAC7	H3K9、H3K27	EC	Inhibit endothelial cell proliferation and angiogenesis
		HDAC9	H3K9、H3K27、H4K12	Mø	Promote M1 polarization and inhibit cholesterol efflux activate vascular inflammation and promote plaque instability
	Class Ⅱb	HDAC6	H3K9、H4K8	EC, Mø, VSMC	Aggravate endothelial dysfunction target the NF-κB/NLRP3 pathway and inhibit macrophage pyroptosis reduce VSMC proliferation and neointima formation
		HDAC10	H3K9、H4K12	Mø	Inhibit macrophage autophagy and promote lipid accumulation in foam cells
	Class III	SIRT1	H3K9、H3K18、H4K16	EC, Mø	Enhance NO bioavailability; protect endothelial function via the AMPK/cortactin axis regulate the expression of lipid phagocytosis and efflux genes, and promote cholesterol efflux
		SIRT3	H3K9、H4K16	EC, VSMC	Reduce mitochondrial ROS production and inhibit cellular senescence
		SIRT6	H3K9、H3K56、H4K16	EC, Mø	Prevent ECs senescence inhibit IL-6 transcription in macrophages and alleviate inflammation
		SIRT7	H3K18、H3K27	VSMC	Promote VSMC proliferation and neointima formation
	Class IV	HDAC11	H3K9、H4K12	EC, Mø	Promote intimal injury Inhibit the transcription of anti-inflammatory gene IL-10 and promote M1 polarization

### 2.1 Endothelial cells: regulation of barrier function and endothelial–mesenchymal transition

Damage to the arterial intima induced by various risk factors and endothelial dysfunction is recognized as a critical initiating event in the development of atherosclerosis. Under physiological conditions, endothelial cells (ECs) maintain vascular homeostasis by producing vasodilator and vasoconstrictor molecules, such as endothelin and nitric oxide (NO), to regulate vascular tone, facilitate vascular repair and preserve endothelial barrier integrity. However, exposure to cardiovascular risk factors alters endothelial permeability and triggers ECs to express adhesion molecules and chemokines, which exacerbates cholesterol-laden low-density lipoprotein (LDL) infiltration and leukocyte rolling and adhesion to the intima (Xu et al., 2021b). The accumulation of oxidized low-density lipoprotein (ox-LDL) in the arterial intima aggravates endothelial inflammation and further induces histone modifications. Studies have shown that HDAC2 protects against endothelial dysfunction induced by ox-LDL and that the number of atherosclerotic plaques in mice overexpressing HDAC2 is significantly reduced. Further investigations revealed that ox-LDL inhibits HDAC2 expression in endothelial cells, promoting acetylation of arginase 2 (Arg2), which in turn reduces NO production and impairs endothelial function (Pandey et al., 2014; Hori et al., 2020). HDAC1 and HDAC2 cooperatively repress VCAM-1 expression by inhibiting the acetylation of STAT3 at lysine 685 and reducing the methylation of the GATA6 promoter, which alleviates atherosclerotic lesion formation (Hu et al., 2021). Histone deacetylases are closely related to endothelial‒mesenchymal transition (EndMT), which is a key factor in stabilizing atherosclerotic plaques. Studies have revealed that HDAC3 expression is upregulated in the aortas of atherosclerotic mice and human umbilical vein endothelial cells (HUVECs) stimulated with tumor necrosis factor-α (TNF-α) or interleukin-1β (IL-1β). HDAC3 inhibition via siRNA or the specific inhibitor RGFP966 suppresses EndMT and inflammation in TNF-α/IL-1β-stimulated HUVECs (Chen et al., 2021). Subsequent studies have shown that HDAC3 influences the differentiation of endothelial progenitor cells and that the overexpression of HDAC3 leads to increased Akt phosphorylation and kinase activity, thereby promoting plaque formation and vascular rupture in *ApoE* ^*−/−*^ mice (Zampetaki et al., 2010). Additionally, HDAC3 protects against atherosclerosis by inhibiting inflammation by inactivating NF-κB/p65 through the upregulation of miR-19b-mediated PPARγ in ox-LDL-treated HUVECs (Wang et al., 2021c). These findings demonstrated that HDAC3 serves as an essential pro-survival molecule with a critical role in maintaining endothelial integrity. Among the Class II histone deacetylases, HDAC4 expression and phosphorylation are markedly upregulated after stroke, and suppression of HDAC4 phosphorylation inhibits tube formation and the expression of genes downstream of HIF-VEGF signaling in an endothelial cell hypoxia model (Liu et al., 2017). A recent study revealed that HDAC6 is recruited to the VEGF gene promoter in ox-LDL-treated HUVECs, enhancing H3K9 deacetylation and vascular endothelial cell injury (Kai et al., 2021). Similar studies have shown that endothelial HDAC6 expression and activity are upregulated, whereas cystathionine γ-lyase (CSEγ) mRNA expression and protein abundance are reduced in ox-LDL-exposed HAECs, thereby reducing the production of H2S and impairing cardiovascular homeostasis (Leucker et al., 2017). Additionally, endothelium-specific HDAC9 knockout in mice significantly suppressed EndMT and reduced atherosclerotic plaque formation. Furthermore, these mice presented a more favorable plaque phenotype, with decreased plaque lipid content and increased fibrous cap thickness (Lecce et al., 2021). Mattagajasingh et al. reported that SIRT1 preserves EC function by targeting the calmodulin-binding domain of nitric oxide synthase (eNOS) at lysines 496 and 506, increasing eNOS activity and NO bioavailability (Mattagajasingh et al., 2007). One study revealed that SIRT1 is poorly expressed in AS and that LincRNA-p21 competitively binds to miR-221, inducing the SIRT1-mediated deacetylation of *Pcsk9*, which promotes endothelial cell tube formation and inhibits the development of AS (Wang et al., 2021a). Studies have shown that SIRT6 suppresses EC pyroptosis by deacetylating apoptosis-associated speck-like protein (ASC), thus inhibiting its interaction with NLRP3 and preventing the progression of AS (Huang et al., 2024). Moreover, SIRT6 also activates the MAPK signaling pathway, promoting apoptosis and suppressing proliferation in ox-LDL-induced HUVECs (Xu et al., 2021c). Although SIRT6 has been reported to be a molecule with anti-atherosclerotic effects, studies have also revealed that SIRT6 has a condition-dependent effect and plays different roles in the functions of HUVECs. Under hypoxic conditions, SIRT6 promotes plaque angiogenesis by activating HIF-1α, whereas under oxidative stress conditions, it binds to the promoter of catalase and reduces H3K56 acetylation, leading to reactive oxygen species (ROS) production and exacerbating neovascular injury (Yang et al., 2021). The HAT p300 exerts dual effects on endothelial cells in atherosclerosis that change with the environment. On the one hand, ox-LDL induces p300-mediated acetylation of the IL-8 and MCP-1 promoters, thereby increasing chemokine expression and leukocyte recruitment (Dje N'Guessan et al., 2009). On the other hand, p300 promotes H3K27 acetylation at the SOCS1 promoter, increasing the transcription of SOCS1 and inhibiting JAK/STAT signaling to reduce endothelial cell damage and mitochondrial dysfunction (Xue and Deng, 2024).

### 2.2 Macrophages: modulation of polarization, foam cell formation, and plaque stability

Macrophages play a crucial role in the process of atherosclerotic lesions. In the early stage of AS, macrophages engulf cholesterol and transform into foam cells, which continuously accumulate to form fat streaks. In the advanced stage, activated macrophages secrete MMPs, causing the fibrous cap of the plaque to thin. Moreover, the apoptosis of macrophages promotes the expansion of the necrotic core of the plaque and enhances the instability of the plaque. Further clarification of the mechanisms leading to macrophage activation and foam cell accumulation is vital for preventing plaque formation and rupture. Studies have reported that histone acetylation can regulate key processes, such as the polarization of macrophages, lipid metabolism, and apoptosis, which play significant roles in plaque formation (Ouimet et al., 2011). A study revealed that HDAC2 deficiency in *LDLR* ^*−/−*^ mice reduces the aortic plaque area and macrophage content. Mechanistically, histone H3 acetylation and MKP-1 activity are increased, and the recruitment of activated macrophages is reduced in HDAC2-deficient monocytes, indicating that HDAC2 promotes atherogenesis via monocyte/macrophage activation (Wang et al., 2021d). Furthermore, myeloid-specific HDAC3 knockout promotes collagen deposition in atherosclerotic lesions and directly increases the acetylation of TGF-β1, driving the transformation of macrophages toward a profibrotic phenotype and an anti-inflammatory phenotype, suggesting that HDAC3 may serve as a potential novel therapeutic target for cardiovascular diseases (Hoeksema et al., 2014). The regulation of histone acetylation is complex. Studies have shown that histone deacetylase 1 (HDAC1) and the histone methyltransferase SUV39H1 can simultaneously bind to the MCP-1 promoter region, decreasing the degree of H3/H4 acetylation and H3K9 methylation, thereby reducing the expression of MCP-1 and inhibiting the recruitment of monocytes to atherosclerotic lesions (Jia et al., 2019). Genome-wide association studies revealed that HDAC9 is highly expressed in atherosclerotic plaques and promotes M1 macrophage polarization. Mechanistically, HDAC9 represses H3K9 acetylation at the promoters of the cholesterol efflux transporters ABCA1 and ABCG1 and the M2 polarization regulator PPAR-γ, which inhibits cholesterol efflux and enhances pro-inflammatory cytokine production, exacerbating foam cell formation and plaque growth (Cao et al., 2014). Additionally, HDAC6 modulates p65 acetylation and *Nlrp3* transcription, reducing pyroptosis-mediated macrophage inflammation and attenuating atherosclerosis (Xu et al., 2021a). In addition to the role of deacetylases, p300 and HAT1 are recruited to the NADPH oxidase 5 (Nox5) promoter, increasing H3K27ac and H3K9ac. This upregulates Nox5 expression, increasing ROS production and promoting pro-inflammatory macrophage activation (Vlad et al., 2019).

### 2.3 Vascular smooth muscle cells: control of phenotypic switching and proliferation

Vascular smooth muscle cells (VSMCs) undergo a phenotypic switch from a contractile state to a synthetic or proliferative state in atherosclerosis, promoting plaque growth and neointimal vessel remodeling. Studies have shown that histone acetylation tightly regulates this switch and the proliferation of VSMCs caused by vascular injury (Findeisen et al., 2011). Recent studies have revealed that HDAC1 is a key regulator of the VSMC proliferation phenotype and that knockdown of HDAC1 significantly reduces the viability and migration of SMCs by suppressing the PKD1-mTOR signaling pathway under quiescent conditions and PDGF-BB treatment (Kwon et al., 2016). Moreover, downregulation of HDAC1 inhibits media degeneration and preserves elastic fiber integrity in a mouse model of thoracic aortic dissection (TAD) (Sun et al., 2020). The overexpression of HDAC1 enhances the inhibitory effect of miR-224-3p on FOSL2 and inhibits the progression of atherosclerosis by deacetylating HIF-1 (Wang et al., 2021b). In contrast, HDAC1 has been shown to mediate homocysteine-induced atherosclerosis by reducing the acetylation level of histone H3 at lysine 9 (H3K9ac), leading to the accumulation of total cholesterol (TC), free cholesterol, and triglycerides and accelerating the progression of atherosclerosis (Zhao et al., 2017). HDAC3 directly interacts with and deacetylates serum response factor (SRF), a critical transcription factor for VSMC contractile gene expression, promoting VSMC phenotypic switching to a synthetic state and neointimal hyperplasia. Inhibition of HDAC3 with specific inhibitors significantly reduces SMC proliferation and neointimal formation after mouse carotid artery injury (Zhong et al., 2024). The excessive activation phenotype of SMCs is an important pathological basis for vascular calcification and is closely related to the occurrence and development of atherosclerosis. HDAC4 expression is increased in early calcified vessels and has been reported to be involved in the proliferation, migration and inflammatory response of SMCs (Lee et al., 2008; Gu et al., 2019). HDAC4 affects PDGF-BB-induced SMC proliferation and migration via ROS generation in a Ca^2+^/calmodulin-dependent protein kinase-dependent manner, activating the p38 MAPK‒HSP27 signaling pathway. This may lead to neointimal hyperplasia and mediate the development of hypertension in spontaneously hypertensive rats (Usui et al., 2014; Abend et al., 2017; Zheng et al., 2019). In addition, HDAC5 has been identified as a pro-inflammatory molecule in VSMCs that mediates Nox4-dependent ROS generation and PI3K/AKT signaling activation (Pietruczuk et al., 2019). Malhotra et al. reported that HDAC9 is also associated with abdominal aortic calcification and that the overexpression of HDAC9 promotes calcification and reduces contractility in human aortic VSMCs (Malhotra et al., 2019). Aging is the foremost risk factor for metabolic syndrome and atherosclerosis, which are the principal causes of cardiovascular diseases (Wang et al., 2025b). Another type of histone deacetylase, SIRT6, has a decreased expression level in human and mouse plaques and is regulated mainly by the ubiquitin ligase CHIP. SIRT6 has been reported to play a key role in regulating the aging of vascular smooth muscle cells and inhibiting atherosclerosis. The overexpression of SIRT6 preserves telomere integrity, delays cellular senescence, and reduces the expression of inflammatory cytokines and changes in VSMC metabolism associated with senescence (Grootaert et al., 2021). A previous study revealed that SIRT6 interacts with the NF-κB RELA (p65) subunit and deacetylates histone H3 lysine 9 (H3K9) at NF-κB target gene promoters, inhibiting NF-κB-dependent gene expression, apoptosis and cell senescence (Kawahara et al., 2009). Furthermore, SIRT1 can also participate in inhibiting the acetylation and expression of p65 and p53, thereby suppressing cellular senescence and atherogenesis (Yan et al., 2021). Studies have shown that SIRT3 reduces the generation of ROS in mitochondria by deacetylating FOXO3a, thereby alleviating oxidative stress and protecting mitochondria from oxidative damage (Velpuri et al., 2024). In addition to its direct deacetylase function on NFATc1 at the K549 site, SIRT3 can also promote the phosphorylation of NFATc1 at the Y270 site induced by FAK. By regulating the acetylation‒phosphorylation interaction, SIRT3 affects the progression of atherosclerosis (Sun et al., 2023).

## 3 Histone methylation in atherosclerosis

Compared with histone acetylation, histone methylation is a more stable epigenetic modification that mainly occurs on lysine (k) or arginine (R) residues of histones H3 and H4. The number of methyl groups added can be divided into mono-methylation (me1), di-methylation (me2), and tri-methylation (me3) groups. The biological effects of histone methylation are complex and depend on the specific residues being modified and the degree of methylation (Zhang et al., 2023a). For example, common methylation sites include H3K4, H3K9, H3K27, H3K36, H3K79 and H4K20. Among them, H3K4me1/2/3, H3K9me1, H3K27me1, H3K36me1/2/3 and H3K79me1/2/3 are usually associated with transcriptional activation, whereas H3K9me3, H3K27me3 and H4K20me2/3 are typical inhibitory marks (Greer and Shi, 2012). The process is dynamically regulated by histone lysine methyltransferases (HKMTs) and histone lysine demethylases (KDMs). Histone methyltransferases include six families of histone lysine methyltransferases (HKMT1-6) and four histone arginine/methionine methyltransferases (PRMT1, 3, 5, 6 and CARM1). Histone demethylases can be roughly categorized into three major classes: lysine-specific demethylases (LSD1 and LSD 2), JmjC domain-containing demethylases (KDM2-6), and PHD finger-containing demethylases (KDM7 and KDM8). By modulating chromatin conformation and DNA accessibility, histone de/methylation controls the transcription of genes involved in vascular cell function, making it a key epigenetic regulator of atherosclerotic initiation and progression (Table 2 for a summary of histone de/methyltransferases in atherosclerosis).

**TABLE 2:** Histone methyltransferase and demethyltransferase in atherosclerosis.

Histone modification enzyme	Class	Name	Targets	Cell type	Function
Histone methyltransferase (HMTs)	KMT1	SUV39H1/2	H3K9	EC, Mø, VSMC	VSMC proliferation and intimal formation increase ROS production and affect endothelial dysfunction transcription of genes related to vascular inflammation
		G9a/GLP/EHMAT1	H3K9	Mø, VSMC	M1 polarization and inflammatory gene transcription regulating plaque heterogeneity
		SETDB1/2	H3K9	Mø	Regulate the expression of inflammatory genes influence the polarization and aggregation of macrophages
	KMT2	SET1A/B	H3K4	EC, VSMC	VSMC proliferation/migration
		MLL1-5	H3K4	EC, VSMC	Regulate the expression of inflammatory genes and affect endothelial cell inflammation
		ASH1L	H3K4、H3K9	Mø	Regulation of genes related to cholesterol excretion and formation of foam cells
		SMYD2	H4K20、H3K36	EC, VSMC	Promote HDAC3 expression VSMC phenotypic transformation and neointimal formation
	KMT4	DOT1L	H3K79	Mø, VSMC	Regulate NF-κB pathway, influence the expression of chemokines and vascular inflammation regulate the expression of lipid synthesis genes, affect macrophage polarization and plaque stability
	KMT5	SET8	H4K20me1	EC	Afftct VCAM-1 expression and endothelial cells-leukocytes adhesion
		SUV4-20H1/2	H4K20me2/3	EC	Afftct VCAM-1 expression and endothelial cells-leukocytes adhesion
	KMT6	EZH1/2	H3K27me1/2/3	Mø, VSMC	Promote inflammatory response and foam cell formation inhibit IGFBP5 expreession and aggravate endothelial inflammation
	KMT7	SET7/9	H3K4	EC, Mø	Promote NO production and regulate endothelial function
	PRMT	PRMT1	H4R3	EC	Activate VEGF transcription to promote angiogenesis excessive activation leads to bleeding within the plaque
		PRMT5	H3R8、H4R3	EC, Mø	Regulated by endothelial H3K9la/H3K18la promotes metabolic reprogramming and angiogenesis
		PRMT4/CARM1	H3R17、H3R26	VSMC	Promote VSMC migration and ECM degradation
Histone demethyltransferase (KDMs)	KDM1	LSD1/KDM1A	H3K4me1/2、H3K9	EC, Mø	Nhance M1 polarization, promote autophagy and NLRP3 activation inhibit the endothelial cell MAPK pathway and inflammatory response
	KDM4	JMJD2A/KDM4A	H3K9me2/3、H3K36me2/3	Mø	Egulate M1 polarization promoting the release of inflammatory factors
	KDM5	KDM5B	H3K4me2/3	EC	Regulated by disturbed flow and aggravates endothelial inflammation enhances the activation mediated by NF-κB
	KDM6	UTX/KDM6A	H3K27me2/3	VSMC	Promote EndMT transformation and accelerate plaque instability
		JMJD3/KDM6B	H3K27me2/3	EC, Mø	Enhance endothelial cell inflammation promote M1 polarization and inflammatory genes transcription
	KDM7	PHF8/KDM7B	H4K20、H3K9	EC	Activate the eNOS gene, promote NO production, and regulate endothelial function

### 3.1 Endothelial cells: regulation of inflammation and the shear stress response

Pro-atherogenic stimuli that trigger endothelial activation and dysfunction are tightly controlled by histone methylation, thereby regulating the expression of pro-inflammatory genes and adhesion molecules and directly influencing leukocyte recruitment and EC barrier integrity. KDM6B was upregulated in LPS-treated HUVECs, reducing the level of the repressive marker H3K27me3 at the promoters of pro-inflammatory genes, such as TNF-α, MMP-9, IL-6, IL-1β and ICAM-1 (Yu et al., 2017). In addition, KDM6B synergizes with NF-κB to increase the recruitment of the activating marker H3K4me3 to these promoters, thereby promoting inflammatory gene transcription and accelerating atherogenesis (Yu et al., 2017). Pro-inflammatory cytokines induce H3K9me3 demethylation at the promoter of AIP1 in ECs and promote AIP1 transcription, leading to increased ROS production and increased cardiovascular disease risk (Li et al., 2020). SUV39H1 expression is downregulated in vascular ECs from obese individuals (Masi et al., 2021), and this reduction disrupts H3K9 methylation, accelerates the endothelial dysfunction induced by ROS and promotes the development of atherosclerosis (Costantino et al., 2019). We found that disturbed flow, a pro-atherogenic force, upregulates the expression of the histone demethylase KDM5B via the Piezo1-ETS-1/c-JUN pathway and that KDM5B then downregulates H3K4me3 at anti-inflammatory gene promoters, exacerbating endothelial inflammation and atherosclerosis (Wu et al., 2024a). In contrast, laminar flow reduces H3K27me3 levels by downregulating the methyltransferase EZH2 and enhances the expression of anti-inflammatory genes, such as eNOS and KLF2, preserving EC homeostasis. These findings highlight the role of histone de/methylation in integrating mechanical signals into endothelial cell function (Xu et al., 2018). Inhibiting the enzymatic activity of LSD1 leads to increased H3K4me3 levels at the MAP2K2 promoter, activating the ERK-MAPK pathway, increasing inflammatory cytokine expression, and further exacerbating the formation of atherosclerotic plaques (Zhang et al., 2023b).

### 3.2 Macrophages: modulating polarization, foam cell formation and inflammation

Histone de/methylations affect the polarization of macrophages into pro-inflammatory M1 macrophages and the formation of foam cells, and dysregulation of this modification drives inflammation and instability in plaques. Research has shown that TRPA1 alters H3K27me3 levels in macrophages and reduces H3K27me3 levels, inducing M1 polarization and enhancing macrophage pro-inflammatory activity (Wang et al., 2020). The EZH2 protein in the polycomb repressive complex 2 (PRC2) is a key enzyme that catalyzes the trimethylation of H3K27 and is associated with gene silencing. In inflammatory-stimulated macrophages, EZH2 promotes the shift of macrophages toward the M1 phenotype and exacerbates chronic inflammation within the plaque by suppressing the expression of anti-inflammatory phenotype genes such as IL-10 and Arg1 via the trimethylation of H3K27 and the formation of an inhibitory chromatin environment that hinders the binding of transcription factors to DNA (Greißel et al., 2016). Additionally, EZH2 inhibits Socs3 expression and activates JAK/STAT signaling, facilitating M1 polarization. Myeloid-specific EZH2 knockout reduces macrophage foam cell formation and decreases inflammatory cytokine release, thereby inhibiting atherosclerotic plaque growth (Neele et al., 2020). Consistent with this, the absence of the myeloid histone H3K27me3 demethylase KDM6B also leads to severe atherosclerosis (Neele et al., 2018). In CD14^+^ monocytes from coronary artery disease (CAD) patients, H3K9me3 and H3K27me3 levels at the MCP-1 promoter are reduced, leading to increased MCP-1 transcription and plasma concentrations (Greissel et al., 2015; Xiao et al., 2018). Studies have shown that SETDB2 is upregulated in M1 macrophages within human and mouse atherosclerotic plaques. SETDB2 knockout enhances vascular inflammation and increases macrophage accumulation in lesions by reducing H3K9me3 levels at proinflammatory gene promoters, promoting excessive inflammation (Zhang et al., 2021). Another study revealed that the H3K9me3 and H3K36me3 demethylase KDM4A directly promotes ox-LDL-stimulated macrophage and increases the transcription of iNOS, TNF-α, MCP-1, and IL-1β, promoting the polarization of macrophages toward the M1 phenotype to accelerate plaque inflammation (Wang et al., 2017). Recent studies have revealed that KDM5B is essential for the NF-κB signaling cascade, the production of cytokines, and the complete activation of macrophages. KDM5B specifically inhibits the expression of IκBα and enhances NF-κB-mediated macrophage activation by removing H3K4me3 at the NF-κB gene locus and reducing chromatin accessibility (Zhang et al., 2023c). Multiple studies have shown that the expression of LSD1 significantly increases in RAW264.7 cells treated with ox-LDL, inhibiting the activation of autophagy through the PI3K/Akt/mTOR signaling pathway (Ambrosio et al., 2017) and further suppressing the NLRP3 activation and inflammatory responses induced by ox-LDL (Zhuo et al., 2019). LSD1 can also shift the polarization of macrophages toward the M1 phenotype in response to hydrogen peroxide stimulation (Tan et al., 2019). Recently, researchers have demonstrated that the joint regulation of LSD1 and SUV39H1 affects the H3K9me2 and H3K9me3 states at the TWIST1 promoter and that this epigenetic modification mediates foam cell formation and enhances the vulnerability of atherosclerotic plaques during hypertension (Sun et al., 2024). The histone H3K79 methyltransferase DOT1L regulates the expression of lipid synthesis-related genes such as the cholesterol-regulatory element binding proteins SREBP1 and SREBP2 by modulating cholesterol, inhibiting macrophage activation within plaques, and promoting plaque stability (Willemsen et al., 2022). Unlike its function in macrophages, DOT1L is specifically expressed in the vascular smooth muscle cells of atherosclerotic lesions, where it induces H3K27me2 modifications in VSMCs, regulating the transcription of NF-κB-1 and -2 and thereby increasing the expression of the chemokines CCL5 and CXCL10 and promoting vascular inflammation (Farina et al., 2022).

### 3.3 Vascular smooth muscle cells: control of phenotypic switching and proliferation

Histone methylations play crucial roles in the phenotypic transformation of VSMCs. In particular, two important methylations, H3K4me3 and H3K27me3, have opposite effects on the expression of VSMC phenotype-related genes. When VSMCs are stimulated by factors such as platelet-derived growth factor (PDGF), histone methyltransferases such as MLL1 are recruited and catalyze H3K4me3 at the promoter regions of synthetic-related genes such as c-myc and cyclin D1, thereby promoting the proliferation and migration of VSMCs. In normal contractile VSMCs, the methyltransferase EZH2 binds to the promoters of synthetic phenotype-related genes, catalyzing H3K27me3, thereby inhibiting the expression of synthetic phenotype-related genes and maintaining the contractile phenotype of VSMCs. In addition to these two epigenetic markers, SUV39H1 regulates the cell cycle progression of vascular smooth muscle cells by catalyzing the trimethylation of lysine at position 9 of histone H3 (H3K9me3). Experimental studies have shown that pharmacological inhibition of SUV39H1 can reduce the enrichment level of H3K9me3 in the p21 promoter region, thereby promoting the expression of the p21 protein and inducing cell cycle arrest in vascular smooth muscle cells (Cherrier et al., 2009). Moreover, the downregulation of SUV39H1 inhibits the migration and proliferation of vascular smooth muscle cells induced by angiotensin II, effectively slowing the formation of new intima after injury. From a mechanistic perspective, SUV39H1 controls H3K9me3 levels at the promoters of p21 and p27Kip1, thereby reducing the abnormal proliferation of smooth muscle cells (Zhang et al., 2019b). A novel epigenetic mechanism revealed that the lysine methyltransferase SMYD2 is overexpressed in neointimal tissue after carotid artery injury. SMYD2 promotes VSMC phenotypic switching and neointimal hyperplasia by catalyzing H3K36me3 at the HDAC3 promoter, increasing SRF and HDAC3 expression (Zhong et al., 2024).

**FIGURE 2:**
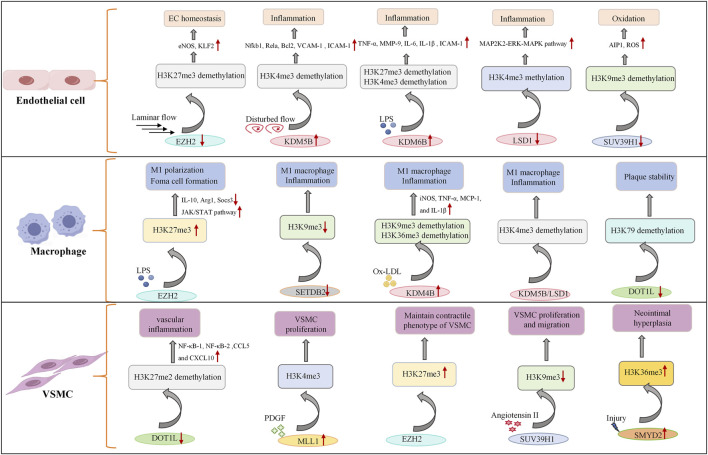
Role of histone methyltransferases and demethylases in endothelial cells, macrophages, and vascular smooth muscle cells. Under the combined influence of cytokines, oxidized low-density lipoproteins and blood flow, the expression of histone methyltransferases and demethylases in vascular cells is regulated, which in turn affects the methylation level of histones within the cells and activates multiple signaling pathways, such as the NF-κB signaling pathway, ERK-MAPK signaling pathway and JAK/STAT signaling pathway, to regulate gene expression and cellular dysfunction. EZH2, enhancer of zeste homolog, histone methyltransferase; KDMs, lysine demethylases; SUV39H1, histone lysine methyltransferase; DOT1L, histone methyltransferase; ↑ indicates upregulation; ↓ indicates inhibition.

During the development of atherosclerosis, the histone de/methylation pattern undergoes dynamic changes and is closely related to the severity of the plaque. In advanced human carotid plaques, immunohistochemistry revealed that the level of H3K4me2 in VSMCs is significantly greater than that in early-stage VSMCs, while the levels of H3K9me2 in VSMCs and inflammatory cells are decreased, and the level of H3K27me2 in inflammatory cells is specifically decreased. In addition, the expression of the histone methyltransferases MLL2 and G9a is upregulated in advanced lesions, contributing to altered methylation patterns (Greissel et al., 2015). Compared with healthy vessels, advanced atherosclerotic lesions exhibit increased H3K9 and H3K27 acetylation in VSMCs, alongside reduced H3K9 and H3K27 methylation in VSMCs and inflammatory cells (Greissel et al., 2015). Notably, Professor Manea et al. recently reported that the transcription of canonical KDM subtypes (including LSD1, KDM2A, KDM3A, KDM4A, KDM5A, and KDM5B) is upregulated in human carotid atherosclerotic samples, further supporting the role of histone demethylation in advanced atherogenesis (Manea et al., 2022). Studies have shown that H3K4 methylation may be associated with the severity of atherosclerotic plaques and that KDM5 family members, in particular, are linked to CVD risk (Mokou et al., 2019). Despite these advances in histone methylation (Figure 2), the crosstalk between different methylation marks and their interactions with other epigenetic modifications in atherogenesis remain incompletely understood and warrant further investigation.

## 4 Histone lactylation in atherosclerosis

**FIGURE 3:**
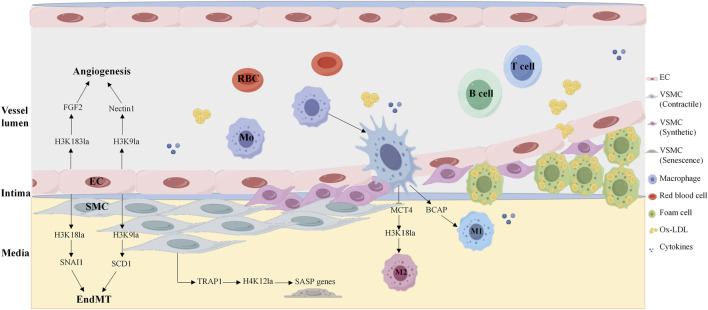
Histone lactylation in key vascular cells in atherosclerosis. Histone lactate modification participates in the development of atherosclerosis by regulating endothelial cell angiogenesis, macrophage anti-inflammatory phenotype polarization, and smooth muscle cell senescence. H4K183la, histone H4 lysine 183 lactylation; H4K9la, histone H4 lysine 9 lactylation; H4K18la, histone H4 lysine 18 lactylation; H4K12la, histone H4 lysine 12 lactylation; SASP, senescence-associated secretory phenotype; EC, endothelial cell; SMC, smooth muscle cell.

Histone lactylation is a novel epigenetic modification first identified by Zhang and colleagues in 2019 and is defined as the covalent attachment of lactyl groups to histone lysine residues with lactate as the donor. This discovery expanded the concept of metabolite-derived acylation and uniquely linked glycolytic flux to epigenetic regulation, as histone lactylation levels are tightly coupled to cellular lactate concentrations (Zhang et al., 2019a). Unlike other histone modifications, lactylation dynamically responds to metabolic states, especially in cells with hypoxia or activated immune cells, where glycolysis is active and the accumulation of lactate is increased. Lactate acts as a signaling molecule by promoting histone lactylation to regulate gene transcription. Histone lactylation is typically associated with transcriptional activation, as lactyl groups neutralize the positive charge of histone lysines, relax the chromatin structure and facilitate transcription factor binding. Histone lactylation shares the same modification site as acetylation, such as the ε-amino group of lysines, explaining why lactylation is also regulated by P300 and HDAC2 (Zhang et al., 2019a). Given its cell-specific roles in ischemia/reperfusion injury, atherosclerosis (Figure 3), and other cardiovascular pathologies, histone lactylation has emerged as a promising yet complex therapeutic target for cardiovascular diseases (Han et al., 2025).

Endothelial cells rely on dynamic metabolic reprogramming to adapt to atherogenic microenvironments, with histone lactylation serving as a critical link between EC metabolism and pathological phenotypes. A study revealed that 77 of 67 proteins presented increased lactylation in the context of increased lactate under hypoxia. The protein Yin Yang-1 (YY1), a transcription factor, is lactylated at lysine 183 (H3K183la), which is regulated by p300. Hyperlactylated YY1 directly binds the promoter of fibroblast growth factor 2 (FGF2), enhancing its transcription and promoting angiogenesis, whereas administration of the p300 inhibitor A485 abrogates YY1 lactylation and suppresses vascularization *in vitro* and *in vivo*, highlighting the therapeutic potential of targeting this axis (Wang et al., 2023). Similar studies have also revealed that a feedback loop between H3K9 lactylation (H3K9la) and HDAC2 in endothelial cells drives VEGF-induced angiogenesis. H3K9la levels are increased in endothelial cells in response to VEGF stimulation and are enriched in the promoter regions of angiogenic genes, such as *Nectin1* and *Tgfbr2*, which activate their transcription to drive angiogenesis. Notably, hyperlactylation of H3K9la inhibits the expression of HDAC2, whereas HDAC2 overexpression reduces H3K9la levels and suppresses the migration, proliferation and tube formation of ECs. Disrupting this loop represents a novel strategy to treat pathological neovascularization (Fan et al., 2024). Further research revealed that lactate induces H3K9 and H3K18 lactylation at the PRMT5 promotor and promotes its expression in neovascular endothelial cells. Mechanistically, SEMA6A recruits RhoA and p300, facilitating p300 phosphorylation and the histone lactylation cycle, thereby inhibiting angiogenesis (Ma et al., 2025b). Histone lactylation also plays an important role in the regulation of EndMT. Research has shown that under ox-LDL stimulation, the histone chaperone ASF1A and histone acetylase P300 together enhance histone H3K18 lactylation, which increases the expression of SNAI1, inducing EndMT and promoting atherosclerosis (Dong et al., 2024). P300 also cooperates with ACSS1 to catalyze H3K9la and transcriptionally activates SCD1, which exacerbates endothelial dysfunction and atherosclerosis (Ma et al., 2025a). These findings reveal a novel mechanism by which energy metabolism and epigenetics regulate atherosclerosis via endothelial cell reprogramming, in which histone lactylation plays a central role. Recent study has shown that protein lactoylation drives EndMT and plaque instability. The lactate transporter aquaporin 9 (AQP9) enhances DRP1-mediated mitochondrial fission via lactate and promotes vimentin and N-cadherin expression while inhibiting VE-cadherin expression, thereby driving endothelial cell mesenchymal transformation and ultimately exacerbating vulnerable carotid plaque formation (Xu et al., 2025). A recent study revealed that IGFBP5 promotes NLRP3 inflammasome-induced EndMT by regulating glycolysis-mediated histone lactylation, accelerating the progression of diabetic nephropathy (Hu et al., 2025).

Macrophage activation is a hallmark of atherosclerosis, and histone lactylation dominates macrophage polarization, governing tissue repair and atherosclerosis progression. Strikingly, the regulation of histone lactylation is stage specific. During the polarization of macrophages toward pro-inflammatory M1 macrophages, histone methylations and histone acetylations are predominant, whereas when macrophages shift to anti-inflammatory M2 macrophages, histone lactylation modifications take the lead, among which the lactylation modification of lysine at position 18 of histone H3 (H3K18la) is the most significant. Monocarboxylate transporter 4 (MCT4) mediates lactate efflux from macrophages, and MCT4 deficiency increases intracellular lactate levels, increasing H3K18la enrichment at the promoters of anti-inflammatory genes and TCA cycle genes, promoting M2 polarization and ameliorating atherosclerotic lesion progression (Zhang et al., 2024). Researchers have reported that histone lactylation plays dual cellular roles in myocardial ischemia‒reperfusion (MI/R) injury. In cardiomyocytes, heat shock protein A12A (HSPA12A) maintains the homeostasis of aerobic glycolysis and H3 lactylation by increasing the ubiquitination and stability of HIF-1α, thereby supporting the survival of cardiomyocytes under hypoxia/reoxygenation (H/R) stress and alleviating reperfusion injury (Yu et al., 2024). In cardiac fibroblasts, lactate accumulation induces lactylation of the serine protease inhibitor A3K (Serpina3k) at lysine 351, which increases Serpina3k protein expression and protein stability, allowing these cells to secrete paracrine signals that activate cardiomyocyte survival kinases and protect against cardiac injury upon reperfusion (Wang et al., 2025a).

The TRAP1-HDAC3-H4K12la axis in VSMCs has been identified as a key regulatory factor in the aging process and plays a crucial role in the development of atherosclerosis. Chen’s group discovered that the overexpression of TRAP1 significantly enhances aerobic glycolysis in VSMCs, leading to an increase in lactate production while specifically inhibiting the activity of HDAC3 and promoting the enrichment of H4K12la in the senescence-associated secretory phenotype (SASP) promoter, promoting the expression of genes such as CCL2, ICAM-1, and IL-6, and exacerbating the aging of VSMCs (Li et al., 2024). Targeted inhibition of TRAP1 or enhancement of HDAC3 activity may be an important strategy for treating aging and cardiovascular diseases.

## 5 Other histone modifications in atherosclerosis

In addition to histone acetylation, methylation and lactylation, other histone post-translational modifications, including ubiquitination, SUMOylation, and phosphorylation, etc. also play context-dependent roles in atherosclerotic pathogenesis.

Histone ubiquitination is a reversible modification characterized by the covalent attachment of ubiquitin molecules to histone lysine residues and is a central player in repairing double-strand breaks (DSBs), a common stress in vascular cells exposed to ox-LDL or inflammation. Approximately 15% of H2A molecules are ubiquitinated, primarily at lysine 119 (H2AK119ub1), whereas only 1%–2% of H2B molecules undergo this modification. H2AK119ub1, which is catalyzed by the RING1A/B and BMI1 subunits of PRC1, is a canonical repressive marker associated with gene silencing and chromatin compaction (Tamburri et al., 2020). It regulates developmental gene expression and maintains cell identity, but its role in AS remains understudied.

SUMOylation is a dynamic modification that attaches small ubiquitin-like modifier (SUMO) proteins to lysine residues, and emerging evidence links SUMOylation to atherosclerosis (Qiu et al., 2017) and diabetes-related complications (Zhong et al., 2025). Recent studies have identified numerous SUMOylated targets, highlighting their emerging regulatory role in the progression of cardiovascular diseases (Du et al., 2021). Our previous work revealed a critical role for non-histone SUMOylation in AS. SUMOylation of HSP90AB1 activates the ERK signaling pathway and promotes monocyte activation and adhesion to ECs, which are the key early steps in atherogenesis (Wu et al., 2025b).

Histone phosphorylation regulates DNA damage repair (Xu et al., 2020), cell cycle progression, and chromatin remodeling (Papamokos et al., 2021). Like acetylation, it neutralizes the positive charge of histones, relaxing chromatin and often promoting transcriptional activation. The phosphorylation of H2AX at Ser139 (γH2AX) is one of the earliest DDR-related histone modifications and leads to persistent DNA damage, cell death, and plaque necrosis (Perez et al., 2020). A recent study revealed that histone H3.3 phosphorylation at serine 31 (H3.3S31) regulates disturbed-flow-induced endothelial inflammation by allowing rapid induction of FOS and FOSB, which are required for inflammatory gene expression (Jin et al., 2025).

Histone O-GlcNAcylation plays a crucial role in vascular dysfunction induced by sustained hyperglycemia (Bolanle et al., 2021). O-GlcNAcylation is a dynamic and reversible post-translational modification of proteins, in which a single N-acetylglucosamine moiety is linked to the hydroxyl groups of serine or threonine residues via an O-glycosidic linkage. O-GlcNAc has been shown to directly modify histones including the mitosis-specific H3Ser10 modifications, and to regulate growth, apoptosis and the cell cycle (Zhang et al., 2011). In a recent study, O-GlcNAcylation was demonstrated to mediate glucose-induced impairment of eNOS activity in endothelial cells: excessive O-GlcNAcylation reduces Akt-mediated phosphorylation and activation of endothelial eNOS, thereby contributing to vascular dysfunction (Masaki et al., 2020). However, other studies have shown that O-GlcNAcylation can suppress acute inflammatory and neointimal responses to arterial luminal injury, indicating that it may also play a protective anti-inflammatory role in the injured arterial bed (Xing et al., 2008).

With the advancement of highly sensitive mass spectrometry (MS) technology, various acylations, including histone crotonylation and succinylation, have been identified (Wang et al., 2025c). Histone crotonylation refers to the transfer of the crotonyl group from crotonyl-CoA to lysine or serine residues and this modification is evolutionarily conserved and typically associated with transcriptionally active chromatin regions (Tan et al., 2011). Dong’s group reported that K305 crotonylation of PKM2 during VSMC phenotypic switching promotes VSMC aerobic glycolysis and intimal hyperplasia (Cao et al., 2025). Multiple studies have indicated that dysregulated lysine crotonylation levels induce cardiomyocyte dysfunction (Ju et al., 2024), vascular oxidative stress (Sun et al., 2025) and arrhythmias (Chen et al., 2023) by altering transcriptional activity and enzyme function. Furthermore, histone H3 lysine 9 crotonylation (H3K9cr) can induce macrophage activation, promote the production of inflammatory cytokines (IL-1β, TNF-α) and chemokines, and exacerbate fibrotic lesions (Zou et al., 2022).

Succinylation involves the covalent attachment of the succinyl group from succinyl-CoA donors to lysine residues (Sreedhar et al., 2020). Succinylation participates in the regulation of cardiovascular diseases by influencing key metabolic pathways, such as the tricarboxylic acid cycle, oxidative phosphorylation, glycolysis, and fatty acid catabolism (Wu et al., 2024b). In microgravity-induced cardiovascular deconditioning, downregulation of SIRT5 increases the succinylation level of endoplasmic reticulum oxidoreductase 1 alpha (ERO1A) at K396, thereby promoting endothelial apoptosis (Pan et al., 2025). Additionally, Chen et al. reported that lactate dehydrogenase A (LDHA) reduces intracellular succinyl-CoA levels in cardiomyocytes, promoting cell proliferation and M2 macrophage polarization, and accelerating cardiac repair after ischemia-reperfusion injury (Chen et al., 2022). Although few direct studies have reported the involvement of rare histone acetylations in atherosclerosis, existing research suggests that metabolic processes and their intermediate products may participate in atherosclerosis by regulating vascular cell functions.

## 6 Targeting histone modifications in the treatment of atherosclerosis

These broad classes of modifying enzymes regulate many different protein modification types, making them attractive targets for drug development (Zheng et al., 2025). Current treatments are limited by suboptimal efficacy and off-target effects, which underscores the need for safer, more effective therapies. In recent decades, epigenetics has become a promising field for developing alternative therapeutic strategies for human diseases, primarily because of the reversibility of epigenetic processes and their high sensitivity to environmental cues (Portela and Esteller, 2010). To date, several classes of epigenetic modulators have been extensively investigated as potential therapies for CVD, including DNA methyltransferase inhibitors (DNMTi), histone deacetylase inhibitors (HDACi), bromodomain and extra-terminal motif inhibitors (BETi), enhancer of zeste homolog 2 (EZH2) inhibitors (EZH2i), and non-coding RNA (ncRNA)-based regulators. Table 3 provides an overview of specific epigenetic drugs that target these mechanisms.

**TABLE 3:** Epigenetic-based drugs and therapy for atherosclerosis.

Epigenetic target	Chemistry/properties	Drug name	Specific target	Mechanism of action
Histone acetylation regulation	HAT inhibitor	Garcinol	p300 HAT、EGR1	Inhibiting the induction of EGR1 transcription and inhibiting VSMCs activation
		MG149 (anacardic acid)	p300 HAT、PCAF	Inhibiting MYST1 and the NF-κB pathway reducing the release of inflammatory factors affecting DNA repair-related Sestrins proteins expression
	HDAC inhibitor	Vorinostat (SAHA)	Class I and class II HDACs	Decreasing the levels of TNF-α, IL-1b, IL-6, and IFN-γ reducing oxidative stress improving endothelial cell dysfunction
		Valproate	Class I HDAC	Mitigating ER stress signaling pathways inhibiting the activity of GSK-3 and blocking the abnormal proliferation of VSMCs
		Panobinostat	Class I/II/IV HDACs	Decreasing hsCRP, sCD40L, MMP-9, IL-6 inhibiting monocytes recrutment and vascular inflammation
		Romidepsin (FK228)	Class I/II HDACs	Inhibit the expression of VCAM-1 decreasing monocytic adhesion to arterial EC
		Tubastatin A (TSA)	HDAC6	Protecting the integrity of the EC inhibiting EC apoptosis and alleviating vascular inflammation
		TMP195 (TFMO2)	Class IIa HDACs	Inhibiting the release of IL-6 and MCP-1 reducing plaque instability
		RGFP966	HDAC3	Inhibiting endothelial-mesenchymal transition inhibiting the inflammation and reducing the expression of IL-6 and MCP-1
		Sodium butyrate	Class I/II HDACs	Inhibiting the proliferation of VSMCs
	BET inhibitor	Apabetalone (RVX-208)	BRD2/3/4	Reducing the expression of LDL-C and hsCRP in the aorta protecting the integrity of the EC
		JQ1	BRD2/3/4	Protecting the function of endothelial cells reducing the release of inflammatory factors
Histone methylation regulation	HMT inhibitor	Chaetocin	SUV39H	Decreasing the expression of MMP9 increasing collagen content and enhancing plaque stability
		GSK126	EZH2	Inhibiting lipid accumulation in macrophages increasing the expression of ABCA1 to promote cholesterol excretion inhibiting the expression of VCAM-1 and reduce the adhesion of monocytes
	KDM inhibitor	GSK-LSD1 2HCl	LSD1	Inhibiting the M1 polarization of macrophages
Histone lactylation regulation		A485	P300/CBP	Inhibiting pathological angiogenesis
Others	Natural compound	Curcumin	p300 HAT、HDAC	Reducing the formation of foam cells inhibiting inflammation
		Resveratrol	SIRT1	Promoting cholesterol excretion reducing the accumulation of foam cells inhibiting the TLR4/NF-κB pathway reducing the release of pro-inflammatory factors

Natural compounds such as garsinol and maslinic acid exhibit histone acetyltransferase inhibitor (HATi) activity, indicating potential modulators of histone acetylation (Legartova et al., 2013). Additionally, histone acetyltransferase activators and other drugs targeting histone modifications are currently in clinical trials (Wang et al., 2015; Hassan et al., 2019). Recent studies indicate that HDAC inhibitors (HDACis) may represent a new class of drugs for the treatment of cardiovascular diseases by influencing post-translational modifications. HDACs regulate multiple biological processes relevant to atherosclerosis, including endothelial cell function, VSMC behavior, cholesterol metabolism, and inflammatory responses. The development of HDAC inhibitors that target the catalytic domain may constitute a new class of cardiovascular therapeutics (Luan et al., 2022). HDACi have already been approved for treating hematological malignancies (Petrella et al., 2011), and vorinostat (suberoylanilide hydroxamic acid, SAHA), romidepsin, belinostat, and chidamide are well-established HDACi (Wu et al., 2025a). In recent years, the academic community has conducted extensive research on the role of histone deacetylases (HDACs) in atherosclerosis, among which research on HDAC1 and SIRT1 is the most in depth. The former exerts a pro-atherosclerotic effect, whereas the latter plays an anti-atherosclerotic role (Yang et al., 2025a). Vorinostat, approved by the FDA for treating cutaneous T-cell lymphoma, inhibits all HDAC classes except Class III sirtuins (Petrella et al., 2011). Trichostatin A (TSA), a pan-HDACi, exacerbates atherosclerosis in *Ldlr* ^*−/−*^ mice by increasing histone acetylation at the promoters of the scavenger receptors CD36, TNF-α, and VCAM-1 while reducing IL-6 and IL-1β expression (Choi et al., 2005; Song et al., 2010). In contrast, a recent study demonstrated that TSA treatment alleviated AS lesions and induced the acetylation of C/EBPα (CCAAT enhancer binding protein alpha), through which it increased PPARγ transactivation and downstream cholesterol transporters and mitigated the induction of the inflammatory cytokines TNFα and IL-1β, suggesting that targeting the C/EBPα/PPARγ axis with HDAC inhibitors has therapeutic potential in slowing the progression of AS and related cardiovascular diseases (Gao et al., 2020). The multiple mechanisms of action of TSA in atherosclerosis may be associated with its non-specific properties, as this drug exerts inhibitory effects on histone deacetylase (HDAC) classes I, IIA, and IIB. Most clinically available HDACi are pan-inhibitors, and inhibitor selectivity must be carefully considered when evaluating HDACi pharmacology to reduce off-target effects (Bondarev et al., 2021). TMP195, a selective class IIa HDACi, alleviates advanced atherosclerosis by suppressing key inflammatory pathways, suggesting a novel strategy to mitigate vascular inflammatory sequelae (Asare et al., 2020; Asare et al., 2025). Romidepsin (FK228), a selective HDAC1/2 inhibitor with anti-inflammatory properties, regulates VSMC proliferation by modulating the deacetylation of transcription factors, such as Kruppel-like factor 5 and CREB-binding protein (Zheng et al., 2011). FK228 inhibits atherosclerosis by enhancing STAT3 epigenetic modification to regulate VCAM-1 expression in HFD-fed *ApoE* ^*−/−*^ mice (Hu et al., 2021). EndMT is another emerging therapeutic target in cardiovascular disease (Jackson et al., 2017). The HDAC3 inhibitor RGFP966 reduces atherosclerotic development in the aortic root of *ApoE* ^*−/−*^ mice by inhibiting EndMT and VSMC proliferation (Chen et al., 2021). Notably, the same type of HDAC can perform distinct functions via different pathways. For example, HDAC1 both enhances miR-224-3p expression to inhibit atherosclerosis (Wang et al., 2021b) and represses miR-34a to promote atherosclerosis (Li et al., 2018). When developing inhibitors, it is also necessary to consider the dual mechanism of HDAC-dependent and HDAC-independent processes.

Histone methylation is often associated with transcriptional repression, and histone methyltransferase inhibitors (HMTi) such as temsirolimus, have demonstrated clinical efficacy in anti-tumor therapy by inhibiting aberrant histone methylation and regulating gene expression (Marzochi et al., 2023). Compared with other epigenetic inhibitors, HMTi has been studied less extensively in cardiovascular disease. Recent evidence indicates that the H3K27me3 methyltransferase EZH2 is involved in autoimmune inflammation and that targeting H3K9me2/3 and H3K27me3 may serve as an effective strategy for treating chronic inflammatory diseases (Zhang et al., 2018). GSK126 is a potent and highly selective EZH2 inhibitor that exerts anti-inflammatory and protective effects on psoriatic arthritis. In advanced atherosclerotic plaques, IGFBP5 expression is significantly downregulated due to epigenetic silencing by H3K27me3, and GSK126 can upregulate IGFBP5 to trigger anti-inflammatory responses, thereby inhibiting monocyte adhesion to ECs (Xu et al., 2018). Additionally, GSK126 not only suppresses H3K27me3 modification but also significantly reduces pro-inflammatory gene expression at both the mRNA and protein levels. Similar studies have shown that GSK126 effectively retards atherosclerosis progression by reducing macrophage foam cell formation and monocyte adhesion (Wei et al., 2021). In addition to GSK126, targeted therapies against SUV39H1 and DOT1L may also help delay atherosclerosis progression (Willemsen et al., 2022; Yang et al., 2025b), and multiple EZH2 inhibitors are currently in clinical trials.

Given that DNA methylation is often accompanied by histone deacetylation, combining DNA methyltransferase inhibitors with HDACi may become a mainstream strategy for treating inflammatory diseases. Notably, metformin, a commonly used anti-diabetic drug, has beneficial effects on diabetes-related atherosclerosis via multiple epigenetic modifications, particularly during aging (Giordo et al., 2023). Statins are clinically used HMG-CoA reductase inhibitors that not only effectively control atherosclerotic inflammation but also partially reverse ox-LDL-induced pathological effects. They reduce histone H3 and H4 acetylation levels and inhibit histone H3 phosphorylation at the promoters of pro-inflammatory genes (Dje N'Guessan et al., 2009). In the pathogenesis of atherosclerosis, histone modifications may not only trigger disease initiation but also serve as biomarkers for assessing disease severity and progression. Therefore, therapeutic approaches targeting epigenetic modifications can target specific pathogenic genes and pathways precisely, enabling more accurate personalized treatment. However, given the potential clinical risks of existing chemotherapeutic drugs and emerging agents that target epigenetic silencing, cell type-specific therapies may represent a new direction for atherosclerosis treatment, but further investigations into the underlying mechanisms are needed.

## 7 Conclusion

Histone modifications are key components of epigenetic regulation, play a central role in deciphering the functions and regulatory mechanisms of physiological and pathological processes, and provide critical insights into disease progression. In this review, we systematically summarize the mechanism of action of histone post-translational modifications related to vascular cell function in atherosclerosis, provide new ideas for the treatment of CVD, and explore possible future research directions related to the relationship between epigenetics and cardiovascular diseases.

Mounting evidence has demonstrated that epigenetic modifications play pivotal roles in the initiation and progression of AS. Microarray-based DNA methylation analyses have revealed higher global DNA methylation levels in atherosclerotic patients than in healthy individuals (Bose et al., 2014). Epigenetics exerts bidirectional regulatory effects in AS, it not only modulates the transcription of inflammatory cytokines but also controls epigenetic modifications through reciprocal regulatory mechanisms. In fact, epigenetic modifications impose stringent network regulation and directional guidance on atherosclerotic-related gene expression and functional changes in various vascular cell types. In turn, these epigenetic modifications are also regulated by multiple atherogenic stimuli. Moreover, when using human atherosclerotic tissue or animal models to determine the role of epigenetics in vascular pathology, the dynamic nature of the disease progression and the heterogeneity of tissue cells also need to be taken into account as influencing factors in the mechanism exploration. For example, in the process of inflammatory regulation, histone methylation and acetylation in macrophages play important roles in M1 polarization, whereas histone lactylation plays a dominant role in the initiation of anti-inflammatory responses. (Zheng et al., 2025).

Another critical challenge is that multiple epigenetic alterations often coexist and present either synergistic or competitive antagonism in established disease states, especially when the same amino acid residue is targeted. For example, the histone acetyltransferase p300 can simultaneously catalyze both acetylation and lactylation at histone lysines 18 and 9, thereby influencing endothelial dysfunction and the progression of atherosclerosis. Protein post-translational modifications crosstalk can orchestrate complex interactions among various modifications, influencing protein functions, signaling pathways, and the regulation of protein networks in cardiovascular disease (Song et al., 2025). The crosstalk between these distinct modifications precisely regulates the cellular fate and pathological processes and increases the complexity of the regulatory mechanism. For example, studies have revealed a strong correlation between the expression of H4K12la, H3K27ac and H3K4me3, which can collectively form an aging-associated specific chromatin microenvironment that coordinates vascular smooth muscle cell senescence and atherosclerotic progression (Li et al., 2024). Additionally, acetylation and SUMOylation contribute to the regulation of protein stability and function, and the interplay between phosphorylation and ubiquitination can increase kinase activity, promoting cell survival and dissemination (Kawaf et al., 2024).

These challenges collectively hinder the safe and effective clinical application of epigenetic drugs, such as off-target effects, limitations of drug delivery systems, and the need for cell-type specificity. How to balance these cell-specific epigenetic differences while coordinating common pathological responses remains unclear, and further research is needed to rationally integrate epigenetic modulators into atherosclerotic treatment regimens. Owing to the environmental complexity and compositional heterogeneity of atherosclerotic plaques, the modulation of a single cell type or a single epigenetic modification cannot fully account for the pathogenesis of AS. A deeper understanding of epigenetic network regulatory mechanisms in atherosclerotic pathogenesis has enormous potential translational value.

In summary, histone post-translational modifications are integral to the epigenetic regulation of atherosclerosis, offering both mechanistic insights and therapeutic opportunities. Addressing these conflicting results, epigenetic modification cross-talk mechanisms and translational challenges are essential for fully harnessing the potential of epigenetic research to improve cardiovascular disease diagnosis and treatment.
